# Neuroprotective effects of Shende’an tablet in the Parkinson’s disease model

**DOI:** 10.1186/s13020-021-00429-y

**Published:** 2021-02-06

**Authors:** Xiaoyan Sheng, Shuiyuan Yang, Xiaomin Wen, Xin Zhang, Yongfeng Ye, Peng Zhao, Limin Zang, Kang Peng, Enming Du, Sai Li

**Affiliations:** 1 Nursing Department, Integrated Hospital of Traditional Chinese Medicine, Southern Medical University, Guangzhou, 510315 Guangdong China; 2 Department of Pharmacy, Guangdong Second Provincial General Hospital, Guangzhou, 510317 Guangdong China; 3 The Centre of Preventive, Integrated Hospital of Traditional Chinese Medicine, Southern Medical University, Guangzhou, 510315 Guangdong China; 4 Department of Pharmacy, Integrated Hospital of Traditional Chinese Medicine, Southern Medical University, Guangzhou, 510315 Guangdong China; 5 Department of Pharmacy, The Fifth Affiliated Hospital, Sun Yat-Sen University, Zhuhai, 519000 Guangdong China; 6 Zhengzhou Yihe Hospital of Henan University, Zhengzhou, 450047 Henan China; 7 Henan Eye Institute, Henan Eye Hospital, Henan Key Laboratory of Ophthalmology and Visual Science, People’s Hospital of Zhengzhou University, Henan University, School of Medicine, Henan Provincial People’s Hospital, Zhengzhou, 450003 Henan China

**Keywords:** Shende’an tablet, Parkinson’s disease, Neuroprotection, α-Synuclein, PGC-1α, Nrf2

## Abstract

**Background:**

Shende’an tablet (SDA) is a newly capsuled Chinese herbal formula derived from the Chinese traditional medicine Zhengan Xifeng Decoction which is approved for the treatment of neurasthenia and insomnia in China. This study aimed to investigate the neuroprotective effects of SDA against Parkinson’s disease (PD) in vitro and in vivo.

**Methods:**

In the present work, the neuroprotective effects and mechanism of SDA were evaluated in the cellular PD model. Male C57BL/6J mice were subject to a partial MPTP lesion alongside treatment with SDA. Behavioural test and tyrosine-hydroxylase immunohistochemistry were used to evaluate nigrostriatal tract integrity. HPLC analysis and Western blotting were used to assess the effect of SDA on dopamine metabolism and the expression of HO-1, PGC-1α and Nrf2, respectively.

**Results:**

Our results demonstrated that SDA had neuroprotective effect in dopaminergic PC12 cells with 6-OHDA lesion. It had also displayed efficient dopaminergic neuronal protection and motor behavior alleviation properties in MPTP-induced PD mice. In the PC12 cells and MPTP-induced Parkinson’s disease animal models, SDA was highly efficacious in α-synuclein clearance associated with the activation of PGC-1α/Nrf2 signal pathway.

**Conclusions:**

SDA demonstrated potential as a future therapeutic modality in PD through protecting dopamine neurons and alleviating the motor symptoms, mediated by the activation of PGC-1α/Nrf2 signal pathway.

## Background

Parkinson’s disease (PD) is the second most common neurodegenerative movement disorder, with incidence of approximately 1.5 % of the population aged 65 years and above, which markedly impacts the health-related quality of life. PD is characterized by the relatively selective and progressive loss of dopaminergic neurons in the substantia nigra pars compacta (SNpc) and the ensuing striatal dopamine (DA) depletion, with a resultant motor deficits such as tremor, rigidity and bradykinesia [1]. While extensive research has already been conducted to elucidate mechanisms underlying the pathogenesis of PD, the exact etiology and full regulative network of PD are yet to be revealed. Currently, varied treatments like levodopa or DA agonists focusing on DA replacement are available [2], however, they only provide some relief to motor symptoms without showing reversal effects of PD progression [3]. Novel therapeutic agents that can protect the dopaminergic neurons, stop or reverse the progression, as well as alleviate motor symptoms are in urgent need for PD patients [4].

Growing evidence has suggested that the pathogenesis of PD can be attributed to multifactorial natures, such as excitotoxicity, neuro-inflammation, protein mishandling, mitochondrial dysfunction and oxidative stress [5]. Oxidative stress, induced by enhanced accumulation of intracellular reactive oxygen species (ROS), harmfully affects lipids, proteins and nucleic acids that leads to initiation of death of neurons, serving as a major contributor in PD progression [6]. Mitochondrial electron transport chain is the major source of ROS and the interruption of these members on this chain such as, complex I, can impact cellular redox homeostasis and provoke the overproduction of ROS, resulting in high oxidative stress [7]. α-Synuclein (α-syn) enriched in brain tissue, is another key factor that causes PD. α-Syn can misfold and aggregate into larger neurotoxic species, leading to vesicular DA leakage and increased oxidative stress, which in turn promote protein misfolding and finally neuronal death [8]. Given that the complex mechanisms that lead to PD, the conventional approach in the treatment of PD using single-drug to act on one specific molecular target is not always effective, requiring other effective treatments, e.g., multi-targeting agents that may be sufficient to prevent, stop or reverse neurodegeneration.

As a complementary or alternative approach to the use of pharmacological treatments, various herbal formulations based on Chinese traditional medicine and modern pharmacological theories have also been developed and applied to treat PD. Shende’an tablet (SDA), approved for Hospital Preparation (No. Z20110027) by Guangdong Food and Drug Administration in 2011, is a hospital preparation of Integrated Hospital of Traditional Chinese Medicine, Southern Medical University through modifying the original Zhengan Xifeng Decoction according to over ten years clinical experience and refined production procedure. SDA is composed with ten Chinese herbs, including *radix polygoni multiflori preparata*, *prunella vulgaris*, *caulis polygoni multiflori*, *salvia miltiorrhiza*, *semen ziziphi spinosae*, *scrophularia ningpoensis*, *radix paeoniae alba*, *radix bupleuri*, *cortex albiziae* and *licorice*, which possesses the functions of enriching blood, nourishing liver and tranquilization and can be used to treat patients with neurasthenia, insomnia and dreaminess [9, 10]. Clinically, as some PD patients often suffer from insomnia, we observed improved insomnia and reduced PD symptoms in a patient after the treatment of SDA, suggesting its potential in treating PD [11]. Thus, in the current study, we aim to examine the activity of SDA in PD model and clarify the detailed protective effect and the underlying molecular mechanisms.

Our results showed that SDA not only displayed potent antioxidant activity and inhibitory effects to PC12 cells death induced by 6-hydroxydopamine (6-OHDA), but also exhibited efficient dopaminergic neuronal protection and motor behavior alleviation efficacies in MPTP induced PD mice. SDA was highly efficacious in α-syn clearance in PC12 cells and MPTP-induced PD animal models, probably mediated by the activation of PGC-1α/Nrf2 signal pathway.

## Materials and methods

### Reagents

SDA was obtained from Integrated Hospital of Traditional Chinese Medicine, Southern Medical University (Guangzhou, China); 1-Methyl-4-phenyl-1,2,3,6- tetrahydropyridine (MPTP), G418, Doxycycline (Dox), Rapamycin (Rap), Selegiline (Sele), Chloroquine (CQ), MG132, Hoechst 33,342, DAPI and rabbit anti-α-syn antibody were purchased from Sigma-Aldrich (St. Louis, MO, USA); Unless otherwise specified, all other chemicals and reagents used were of analytical grade.

###  Cell culture

PC12 cells were purchased from the American Type Culture Collection (ATCC, USA) and cultured in Dulbecco’s modified Eagle’s medium (DMEM) supplemented with 10 % heat-inactivated horse serum (HS), 5 % fetal bovine serum (FBS), 100 U/mL penicillin and 100 µg/mL streptomycin (Carlsbad, CA, USA) at 37 ^o^C in a 5 % CO_2_ atmosphere incubator. PC12 cells were transfected with A53T α-syn genes (PC12/α-syn cells) and then stimulated with Dox to overexpress A53T α-syn. PC12/α-syn cells were cultured in DMEM containing 10 % HS, 5 % FBS and 200 µg/mL G418 (Sigma-Aldrich, MO, USA) at 37 ^o^C in a 5 % CO_2_ atmosphere incubator.

### Animals

The male C57BL/6J mice (21–25 g, 10 weeks) and male SD rats (220–250 g) were provided by Guangdong Medical Laboratory Animal Center. Male C57BL/6J mice were used for the preparation of PD model. Serum samples were collected from SD rats. All animals were raised on a 12 h light/dark cycle with ad libitum access to food and water and were housed in clean cages under conditions of 24 ± 1^o^C room temperature and 55 ± 5 % relative humidity. All experimental protocols were conducted according to guidelines of the experimental animal care and use committee of Southern Medical University (Guangzhou, China). The experimental protocols were approved by the Ethics Committee for Animal Experiments of Integrated Hospital of Traditional Chinese Medicine, Southern Medical University.

###  Serum samples

Briefly, 10 male SD rats were randomly divided into two groups. Rats in SDA group were intragastric administrated with SDA (900 mg/kg) twice a day for 5 days. Solvent saline was used in control group. Then on the 6th day, serum samples were collected and analyzed for PC12 cells protection.

### Cell viability

CCK-8 assay was performed in a 96-well plate. Briefly, PC12 cells (1 × 10^4^ cells/well) were incubated with different concentrations of SDA serum samples for 24 h and then exposure to 60 µM 6-OHDA (Sigma-Aldrich, MO, USA) for further 24 h. The absorbance was measured at 570 nm on a microplate reader. Cell viability was expressed as a percentage of the control group.

### Flow cytometer analysis

Annexin V-FITC/PI double staining analysis was used for apoptosis. For experiment group, PC12 cells were treated with 20 % SDA serum for 24 h and then exposure to 60 µM 6-OHDA for further 24 h. After treatment, the cells were collected and stained with Annexin V-FITC/PI and then subjected to flow cytometry analysis (BD Accuri C6). The positive control group was treated with 6-OHDA and blank serum, while the negative control cells were treated with blank serum.

### Hoechst staining assay

PC12 cells were subcultured in a 35 mm confocal dish at a density of 5 × 10^4^ cells/dish and allowed to adhere for 12 h. After washing with PBS for 3 times, the cells were fixed with 4 % polyformaldehyde (PFA), incubated with Hoechst 33,342 (5 mg/mL) for 5 min at 4 ^o^C, and then observed by fluorescence microscope.

###  siRNA transfection

PC12 cells transfected with A53T α-syn genes were stimulated with Dox to achieve the overexpression of α-syn (inducible PC12/α-syn cells), and then these cells were seeded in 6-well plates at a density of 2 × 10^5^ cells/well and allowed to adhere for 12 h. Cells were transfected with Nrf2 siRNA or scrambled siRNA (Santa Cruz, CA, USA) for 48 h using SureFECT transfection reagent according to the manufacturer’s instructions. After induction of Dox (1 µg/mL) for 24 h to induce the expression of α-syn, cells were treated with SDA for another 24 h and then collected for Western blot assay.

### MPTP-induced mice PD model

The mice PD model was constructed by an acute MPTP protocol [12]. Briefly, MPTP, a selective DA neurons neurotoxin, was injected (30 mg/kg/day i.p.) for 5 days to selectively destruct DA neurons and induce experimental Parkinsonism. After 3 days of a resting period, SDA treatment groups (100, 300 and 900 mg/kg), selegiline (10 mg/kg) or equal volume of saline were administered orally twice daily for 2 consecutive weeks.

### Behavior test

At the end of treatment, mice were tested behaviorally (pole, rotarod and open field tests) to evaluate the different aspects of Parkinsonism.

The pole test was used to evaluate the coordination of mice limbs [13]. The pole (length 50 cm, diameter 1 cm) was fixed one 1.5 cm-diameter ball in its upper end and wrapped in gauze to prevent slipping. The trial of mice descending to the bottom platform was performed three successive times with 1 h interval. The time was recorded and average value of three trials was calculated for statistical analysis.

Rotarod test was also used to measure the movement and coordination of PD model mice [14]. Mice were positioned on the rotarod (Anhui Zheng Hua Instrument Co., Ltd., China), and then tested on the revolving rod at the speed of 5 rpm for up to 120 s. The first fell off rod time was recorded automatically and designated as latency. The trial was performed three successive times with 1 h interval and average time was calculated for statistical analysis.

The open field test was performed in an open field apparatus (length 50 cm, width 50 cm, height 40 cm), which consists of clear plexiglass walls and floor. Briefly, mice were placed individually in an acrylic apparatus with a floor, which was divided into equal squares (length 25 cm, width 25 cm), then left to freely explore the arena for 5 min. The movement distance was recorded for statistical analysis [15].

### Tyrosine-hydroxylase (TH) immunofluorescence

Briefly, the brains were collected, fixed with 4 % PFA (v/w = 10:1) overnight at 4 °C, dehydrated with graded sucrose solution (10 to 30 %), OTC-embedded and sliced to make 25 µm coronal sections encompassing the entire SNpc for TH staining. Sections were permeabilized with 0.3 % Triton X-100 for 15 min, and then blocked with 5 % Bovine serum albumin (BSA) in PBS for 1 h at room temperature. And then they were incubated with anti-TH antibody (Millipore, Billerica, MA, USA) overnight at 4 °C, after that the sections were incubated with anti-rabbit secondary antibody conjugated with FITC (Beverly, MA, USA) for 60 minutes at 37 °C. Photographs were taken under a fluorescence microscope (BX51, Olympus Corp, Japan).

### HPLC analysis

Endogenous level of DA and its metabolites (DOPAC, 3,4-dihydroxyphenylacetic acid and HVA, homovanilic acid) were measured using reverse-phase HPLC. Briefly, striatal tissues were dissected rapidly on ice, immediately frozen in liquid nitrogen and stored at − 80 ^o^C for further biochemical analysis. The weighed striatum was sonicated in 0.1 N perchloric acid, the homogenate was centrifuged at 12,000*g* for 10 min at 4 °C, and then 20 µL of supernatant collected was injected into an HPLC-electrochemical detection system equipped with Agilent Eclipse Plus C18 reverse phase column (4.6 × 150 mm). The endogenous level of DA and its main metabolites was expressed as ng/mg tissue weight.

### Western blotting

Samples were homogenized with RIPA buffer containing 1 mM PMSF and 1 % halt phosphatase inhibitor cocktail on ice. After centrifugation, the supernatants were retrieved and protein concentrations were measured with a BCA kit. 30 µg protein per sample was separated by 10–15 % SDS-PAGE and transferred to PVDF membranes. After blocking with 5 % skim milk for 1 h, the membranes were incubated with various primary antibodies overnight at 4 °C and then incubated with secondary antibodies at room temperature for 2 h. The protein bands were detected using an enhanced chemiluminescence plus kit (Amersham Bioscience, Aylesbury, UK). β-Actin was used as the loading control and the immunoreactivity of each protein was performed using Care stream Molecular Imaging Software.

### Statistical analysis

All experimental were analyzed by the GraphPad Prism software 5 (GraphPad, San Diego, CA, USA). One-way ANOVA and Tukey’s multiple comparison test methods were used in the analysis method. The data were expressed as mean ± SEM. *P* < 0.05 was considered to be significant statistically. All experiments were repeated at least three independent times.

## Results

### SDA alleviates 6-OHDA-induced PC12 cell death

**Fig. 1:**
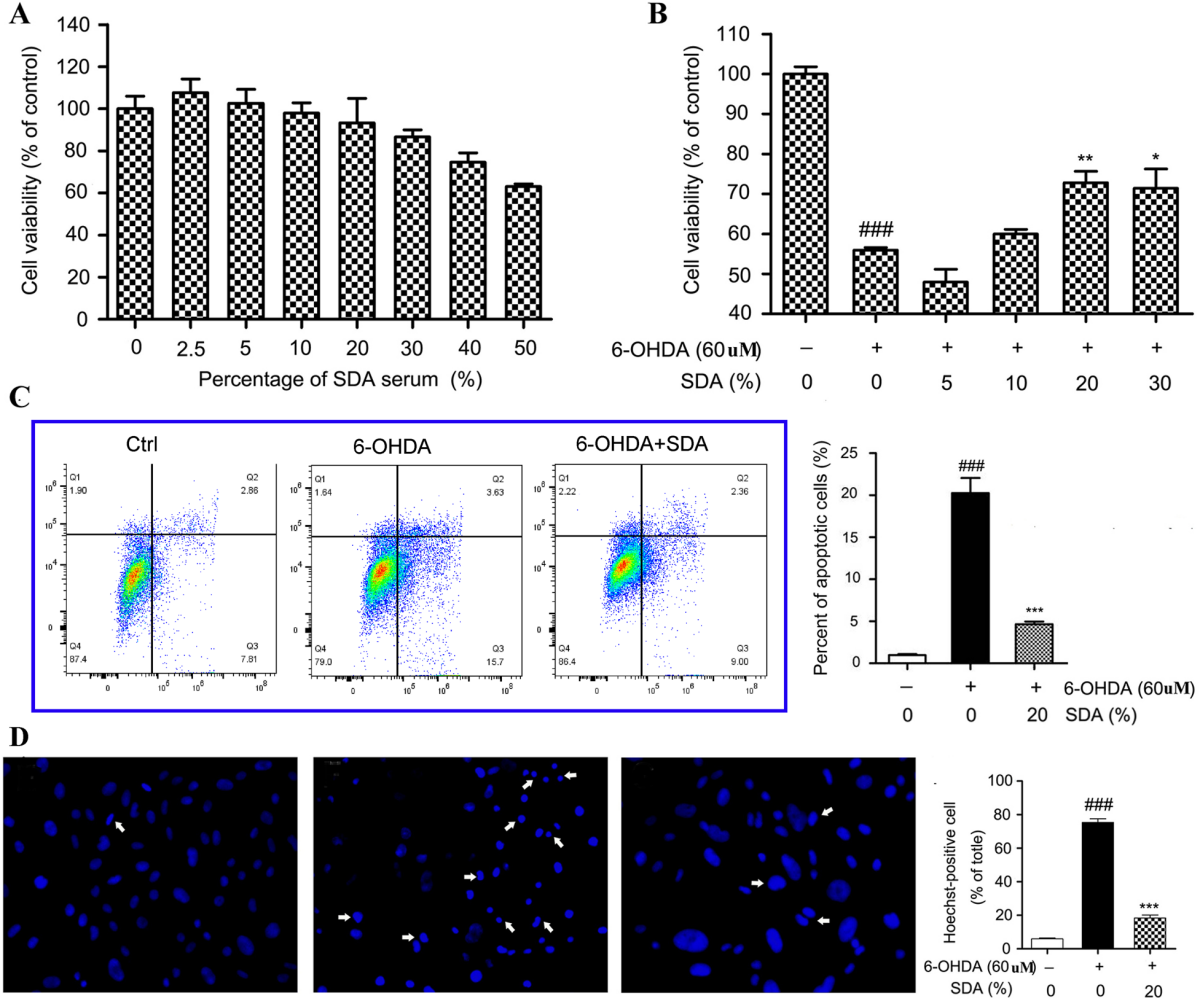
Neuroprotection of SDA rat serum against 6-OHDA-induced toxicity in PC12 cells. **a** The dose-dependent effect of SDA rat serum on the viability of PC12 cells; Neuroprotection of SDA rat serum against 6-OHDA-induced toxicity in PC12 cells was measured by CCK-8 assay (**b**), flow cytometry assay (**c**), and Hoechst staining. **d** The values are presented as the mean ± SEM from three independent experiments (^*###*^ *P* < 0.001 compared to control group; ^*^ *P* < 0.05, ^**^ *P* < 0.01 and ^***^ *P* < 0.001 compared to 6-OHDA group)

PC12 cells treated with SDA rat serum (< 20 %) alone showed nearly the same cell viability compared to untreated controls (Fig. 1a), indicating the nontoxic profile of SDA rat serum at doses below 20 %. 6-OHDA is a widely used parkinsonian toxin, which mimics oxidative stress generation observed in PD and induces degeneration of DA neurons. A significant neurotoxicity was observed and the cell viability was remarkably decreased to 56 % in cells exposed to 60 µM of 6-OHDA for 24 h. In contrast, in the cells pretreated with SDA rat serum followed by exposure to 6-OHDA treatment group, 20 % SDA rat serum afforded the greatest protective effect, which increased the cell survival by 27 % compared to that of 6-OHDA alone (Fig. 1b). The potential neuroprotective effect of SDA on 6-OHDA-induced toxicity was further evaluated by flow cytometry assay and Hoechst staining, and the results indicated that SDA could significantly reverse PC12 cells death induced by 6-OHDA (Fig. 1 c, d). These data strongly suggested SDA provided a solid neuroprotective effect in preventing 6-OHDA-induced PC12 cell death and apoptosis.

### SDA increases the clearance of α-syn via PGC-1α/Nrf2 signaling regulated by UPS pathway

**Fig. 2:**
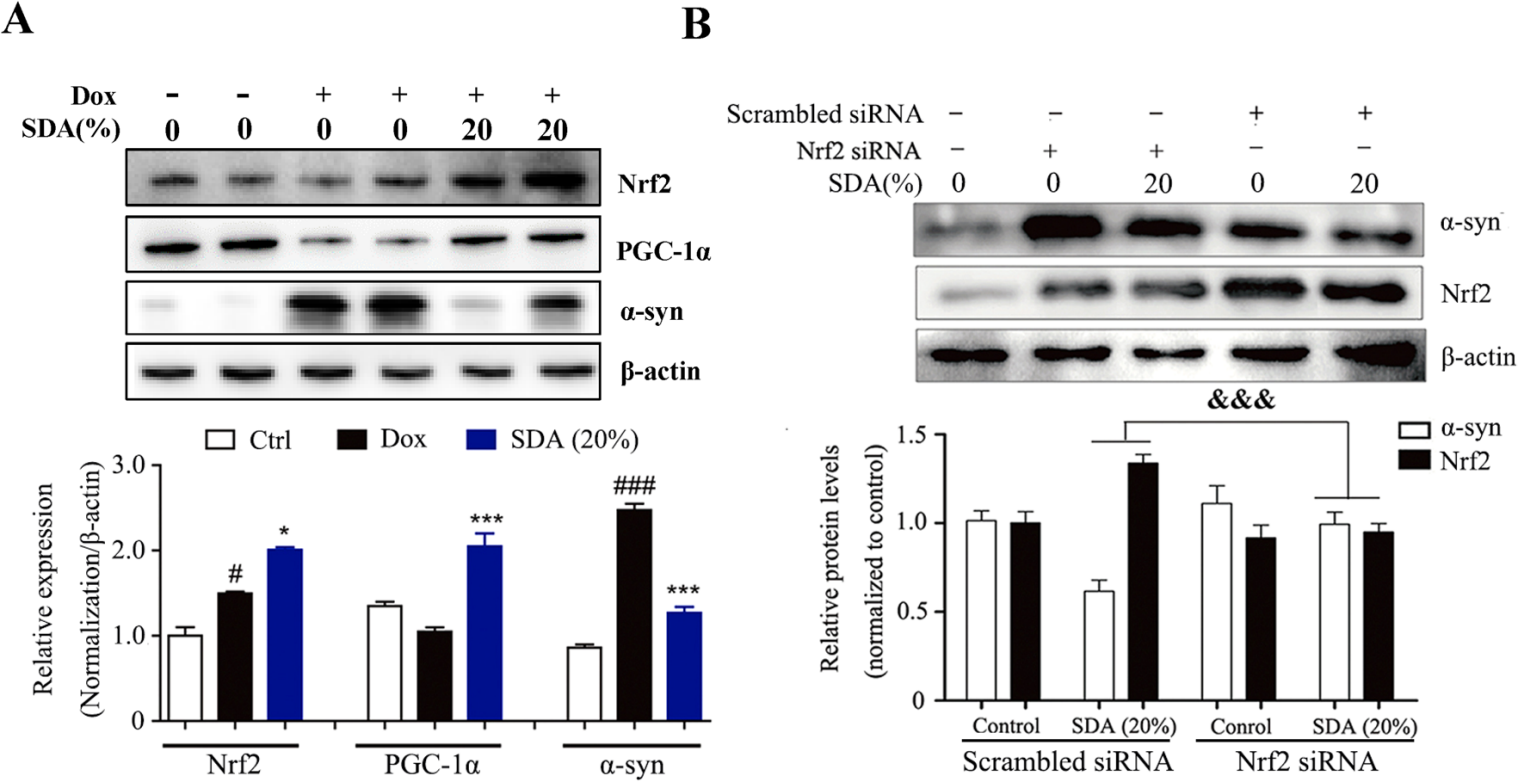
SDA promotes the α-syn clearance through activation of PGC-1α/Nrf2 signaling. **a** Representative immunoblots and densitometry data for α-syn, Nrf2 and PGC-1α in the inducible PC12/α-syn cells treated with doxycycline (Dox) followed by SDA; **b** Representative immunoblots and densitometry data for Nrf2 and α-syn levels in the inducible PC12/α-syn cells transfected Nrf2 siRNA or scrambled siRNA. Data from three independent experiments were expressed as mean ± SEM (^*#*^ *P* < 0.05, ^*###*^ *P* < 0.001 compared to control group; ^*^ *P* < 0.05, ^***^ *P* < 0.001 compared to Dox-treated group; ^*&&&*^ *P* < 0.001)

**Fig. 3:**
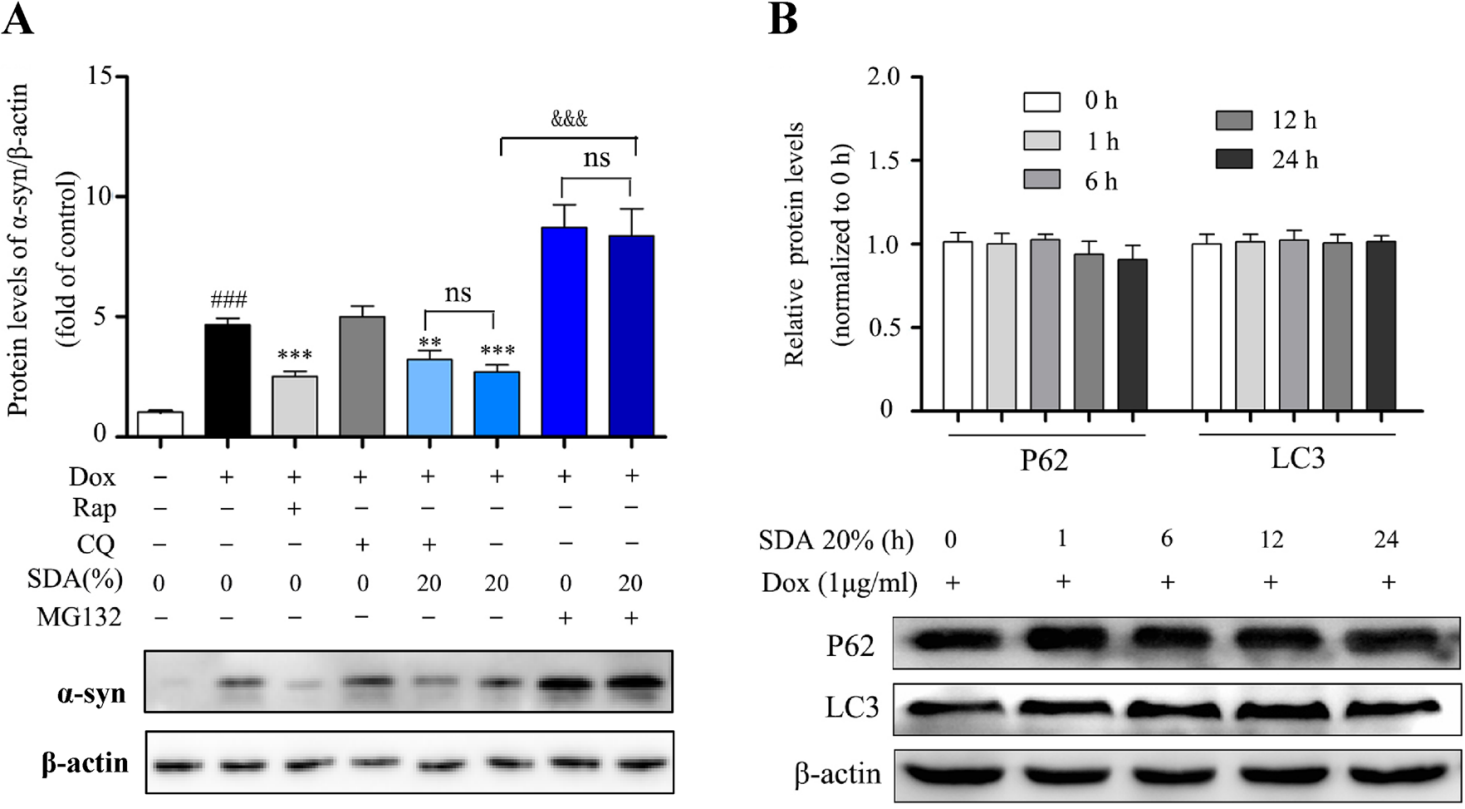
SDA promotes the α-syn clearance regulated by UPS pathway and independent of ALP pathway. **a** Representative immunoblot and quantification of α-syn levels in the inducible PC12/α-syn cells treated with Dox followed by 20 % SDA, 20 µM autophagy inhibitor CQ, 0.7 µΜ proteasome inhibitor MG132 or 0.2 µM mTOR inhibitor Rap for another 24 h; **b** Representative immunoblots and quantification of p62 and LC3 levels in the inducible PC12/α-syn cells treated with Dox followed by SDA. Data from three independent experiments were expressed as mean ± SEM (^*###*^ *P* < 0.001 compared to control group; ^**^ *P* < 0.01, ^***^ *P* < 0.001 compared to Dox-treated group; ^*&*^ *P* < 0.05, ^*&&&*^ *P* < 0.001)

To investigate the potential α-syn clearance of SDA and the underlying mechanisms, the expression levels of PGC-1α/Nrf2 and related protein factors were observed using Western blotting analysis. PC12 cells transfected with A53T α-syn genes (PC12/α-syn cells) were stimulated with Dox to overexpress α-syn, while the expression of α-syn decreased markedly with SDA pretreatment. However, inducible PC12/α-syn cells stimulated with Dox showed no significant change in the protein expression of PGC-1α. Consequently, compensatory increase of Nrf2 was observed after SDA treatment (Fig. 2a). Moreover, Nrf2 knockdown abolished the increased clearance of α-syn upon SDA treatment (Fig. 2b). The ubiquitin-proteasome system (UPS) and the autophagy-lysosomal pathway (ALP) are the two main routes for α-syn clearance [16, 17]. As shown in Fig. 3a, α-syn expression was induced in the stable PC12/α-syn cells, while mTOR inhibitor rapamycin (Rap), a well-established inducer of autophagy, enhanced α-syn clearance. SDA could reduce the expression of α-syn even under the treatment with autophagy inhibitor CQ and no significantly difference of the α-syn expression was observed between the group of CQ/SDA and SDA alone. α-Syn levels was largely increased by treatment with the proteasome inhibitors MG132, without showing significantly statistical differences compared to that of MG132/SDA group. Furthermore, the immunoexpression of the autophagy-related proteins (p62 and LC3) showed no statistically significant differences at different time points after SDA treatment (Fig. 3b). Taken together, these findings illustrated that the clearance of α-syn by SDA was associated with activation of PGC-1α/Nrf2 signal pathway mediated by UPS but not ALP.

### SDA improves motor functions in MPTP-lesioned mice

**Fig. 4:**
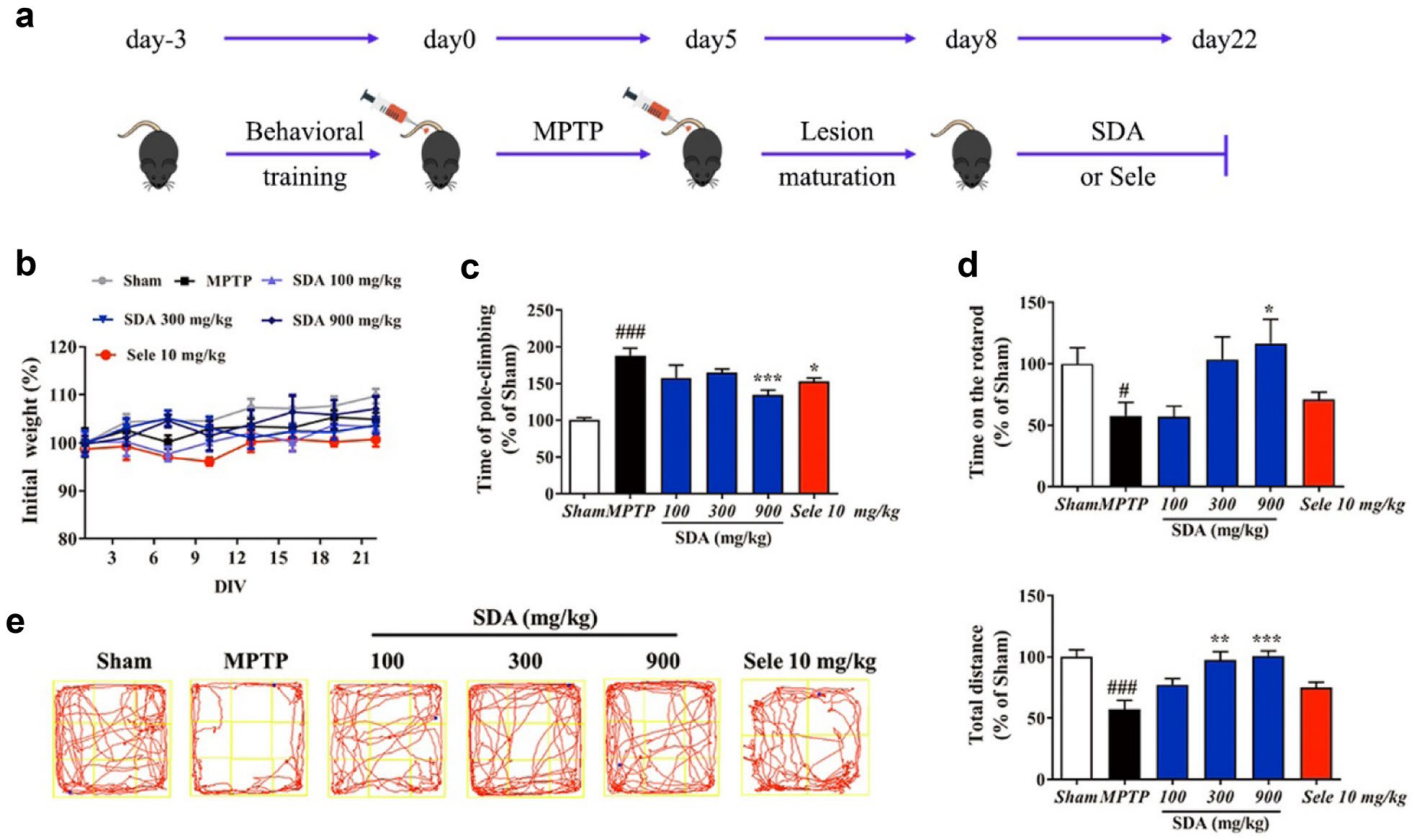
SDA improves motor behavior in MPTP-induced PD mice. **a** A flow chart of MPTP-induced PD animal model. **b** Body weight (g) after administration; **c** Climbing time spent on the pole (% of sham); **d** Time on the rotarod (% of sham); **e** Quantification the total distance in the open field test (% of sham). Data were expressed as mean ± SEM (^*#*^ *P* < 0.05 and ^*###*^ *P* < 0.001 compared to sham group; ^*^ *P* < 0.05, ^**^ *P* < 0.01 and ^***^ *P* < 0.001 compared to MPTP group. n = 6/group)

MPTP, the most frequently used parkinsonian neurotoxin, was applied to constructed PD animal model as shown in Fig. 4a and then used for evaluating motor function improvement and neuroprotective effects of SDA. To investigate whether the impairments of motor performance induced by MPTP could be reversed by SDA, we first evaluated motor performance with the pole test, rotarod test and the open field test. We observed no significant changes in body weight after SDA, MPTP or anti-PD drug selegiline administration (Fig. 4b). Statistical analysis of quantitative data showed that MPTP administration induced a significant increase in pole-climbing time as well as the decrease in rotarod time and total-travelled distance compared with vehicle, suggesting the motor function impairments. Importantly, motor function improvement was observed in high-dose SDA (900 mg/kg) group compared with the MPTP group. Moreover, the observed improvement efficacy of high-dose SDA was somewhat greater than that of anti-PD drug selegiline (10 mg/kg), as indicated in Fig. 4c, e. These data strongly suggested that treatment with high-dose SDA prevented the motor function impairment in PD mice induced by MPTP.

### Reversal of MPTP-induced toxicity in mice by SDA

**Fig. 5:**
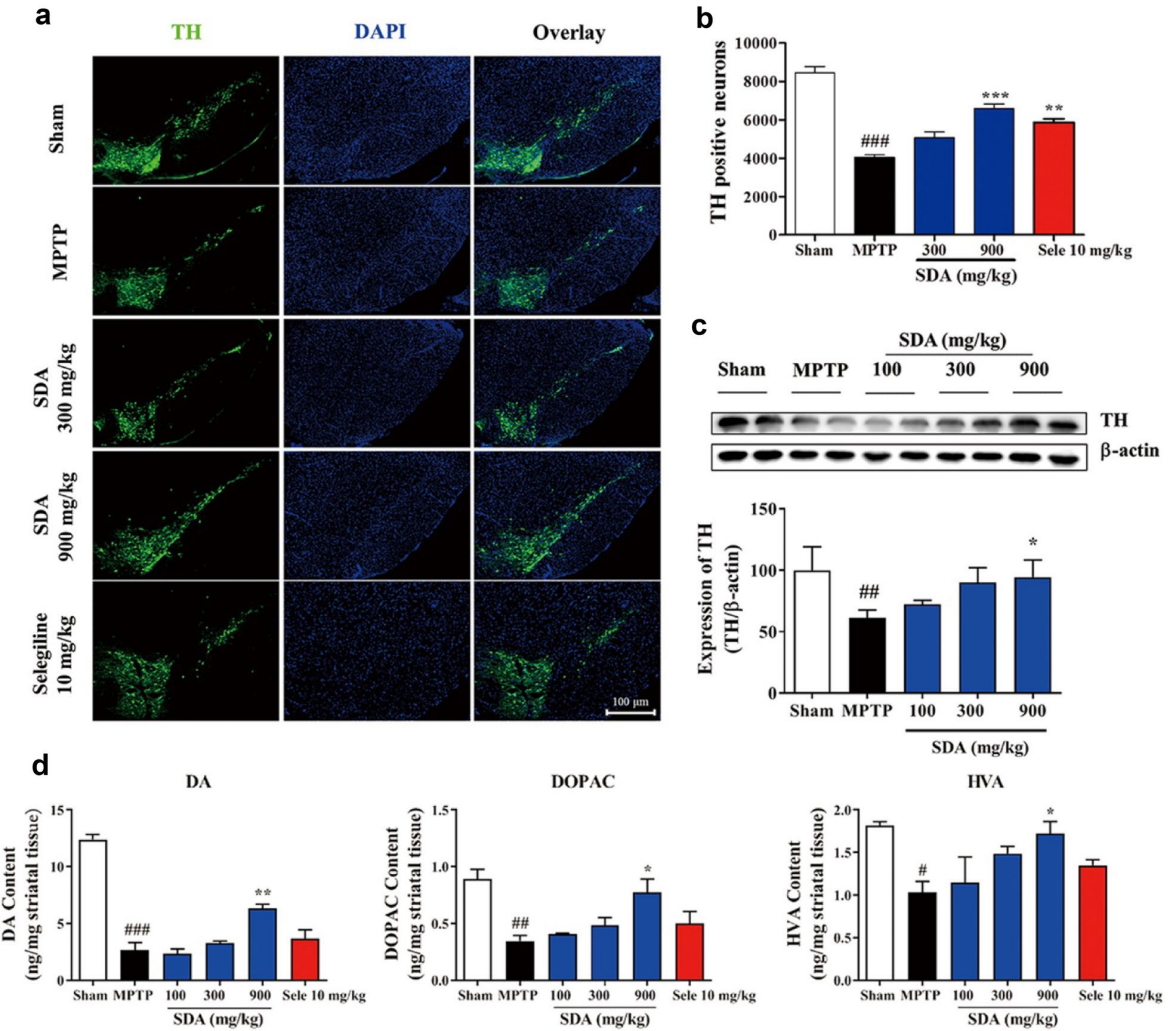
SDA attenuated the Loss of Dopaminergic Neurons and its effect on Striatal DA, DOPAC, and HVA in MPTP-Induced Mice. **a** Representative photomicrographs of TH immuno-staining in the SNpc. Scale bar, 100 µm. **b** The mean number of TH-positive neurons. **c** Western blot assay of TH expression in SNpc. **d** Quantification of DA, DOPAC and HVA (ng/mg) by HPLC in the striatum. Data were expressed as mean ± SEM.  (﻿^##^ *P* < 0.01 and ^###^ *P* < 0.001 compared to Sham group; ^*^ *P* < 0.05, ^**^ *P* < 0.01 and ^***^ *P* < 0.001 compared to MPTP group. n = 3/group)

To evaluate the effect of SDA on dopaminergic neurons loss, TH immunoreactivity was performed and these data confirmed that MPTP induced a significant loss of TH-positive cells compared to vehicle and developed a PD-like behavioral and pathological phenotypes. MPTP administration combined with either SDA or anti-PD drug selegiline treatmnet could prevent the loss of dopaminergic neurons in the SNpc. As shown in Fig. 5a, b, SDA suppressed the loss of dopaminergic neurons in a dose-dependent manner and it was highly efficacious in reversing neurons number in mice at 900 mg/kg compared to MPTP treatment alone. Similar trend was also observed with the clinically used anti-PD drug selegiline at 10 mg/kg, which was as effective as SDA at 300 mg/kg. Western blotting confirmed that the expression of TH protein significant decreased in SNpc, which could be significantly ameliorated with 900 mg/kg SDA treatment (Fig. 5c).

To further assess the effect of SDA on DA metabolism, levels of DA and its metabolites 3,4-dihydroxyphenylacetic acid (DOPAC) and homovanillic acid (HVA) in the striatum were measured by reverse phase HPLC. MPTP treatment significantly reduced the levels of DA, DOPAC and HVA in the striatum of mice, all of which were markedly increased by SDA at 900 mg/kg (Fig. 5d). Similarly, 10 mg/kg selegiline also attenuated MPTP-induced decrease of striatal DA and its metabolites levels without showing statistical significance. The results of these evaluations indicated that SDA can effectively reverse the neurological damage in PD mice induced by MPTP.

### SDA upregulates PGC-1α/Nrf2 expression in the SNpc of MPTP-induced mice

**Fig. 6:**
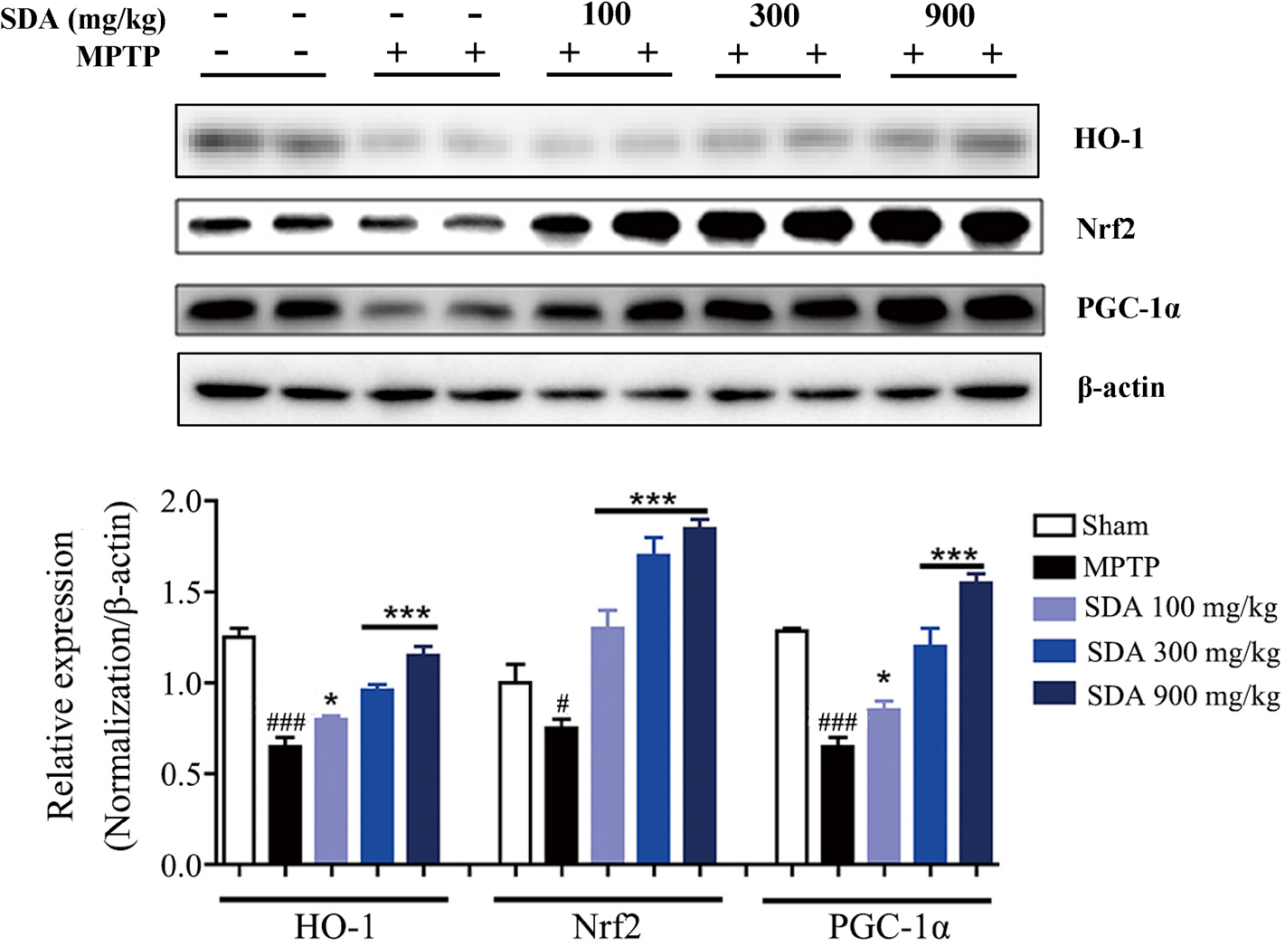
SDA activates PGC-1α/Nrf2 pathway to prevent neurodegeneration in MPTP-induced mice. Representative immunoblots and quantification of HO-1, Nrf2 and PGC-1α in the SNpc of MPTP-induced mice. Data were expressed as mean ± SEM. ^#^ *P* < 0.05 and ^###^ *P* < 0.001 compared to sham group; ^*^ *P* < 0.05 and ^***^ *P* < 0.001 compared to MPTP group. n = 3/group

Finally, to establish a possible mechanism of SDA in reversing the MPTP-induced neurological damage, we assessed the expression of HO-1, PGC-1α and Nrf2 in the SNpc of MPTP-induced mice. As shown in Fig. 6, MPTP-mediated decrease in the expression of HO-1, PGC-1α and Nrf2 levels was observed and this effect was dose-dependently abolished after SDA administration, indicating that neuroprotective effects of SDA was associated with activation of PGC-1α/Nrf2 signal pathway.

## Discussion

Oxidative stress has been considered as a major contributor in PD progression as PD patients have shown accumulations of oxidative damage. The dysfunctioned mitochondrial is a major source of ROS, which was produced as byproduct in mitochondrial electron transport chain. Excessive ROS and increased oxidative stress can further attack biomolecules such as proteins, lipids, and nucleic acids in cells, leading to apoptosis [18–22]. 6-OHDA, a hydroxylated DA analogue, is a commonly used neurotoxin to study PD by initiating the generation of oxidative stress via its autooxidation and subsequent hydrogen peroxide generation [23–25]. In this study, we observed a significant increase of PC12 cells death induced by 6-OHDA, and this effect can be remarkably reversed by SDA treatment via its ability in reducing the oxidative stress in this in vitro model.

One of the characteristic pathologies of PD is the α-syn accumulation and aggregation [21, 26, 27]. A53T missense mutations in the α-syn gene have been proved to increase the α-syn expression [28]. A growing body of evidence suggests that a close connection between α-syn, oxidative stress, and mitochondrial dysfunction in PD progression [21]. Damages caused by oxidative stress contribute to α-syn aggregation, while α-syn overexpression also enhances the ROS production [29, 30]. In our in vitro model, PC12/α-syn cells were stimulated with Dox to overexpress α-syn which could be efficiently cleared by SDA mediated by the activation of PGC-1α/Nrf2 signaling. α-Syn clearance caused by SDA could be significantly reversed by the pretreatment with proteasome inhibitors MG132 in the stable doxycycline-inducible PC12/α-syn cells, but not disturbed by autophasy inhibitor CQ, indicating a mechanism mediated by UPS but not the ALP.

The culture-based PC12 cell survival assay represents a simplified experimental system that may not correctly reflect the complexity of PD. Therefore, to extend these investigations to in vivo conditions and in order to clarify their underlying mechanism [31], in the present study, MPTP-induced PD animal model was used for further evaluation. MPTP is a mitochondrial complex I inhibitor, which can be converted to the toxic metabolite 1-methyl-4-phenylpyridinium (MPP^+^) by the monoamine oxidase (MAO) enzyme in astrocytes. Thereafter, MPP^+^ is released into the extracellular space and then selectively taken up to the dopaminergic nerve terminals, resulting in the oxidative damage of dopaminergic neurons. In addition, MPP^+^ can accumulate within mitochondria and cause mitochondrial dysfunction by inhibiting complex I of the electron transport chain, which can also provoke the overproduction of ROS [23]. Due to the ability of readily passing the blood brain barrier, MPTP exposure can replicate the neuropathological and behavioral features of PD. In the present study, a dose of 30 mg/kg/d of MPTP for 5 days significantly impaired motor behavior, decreased nigral DA neurons and deplete striatal DA and its metabolites (DOPAC and HVA) levels in the SNpc, indicating a successful model of PD. Remarkably, these PD-like symptoms could be reversed by SDA at a dose of 900 mg/kg/d after 2 weeks treatment, indicating its potential in protecting the dopaminergic pathway and improving the behavioral deficits in PD animals. In this study, SDA (300 mg/kg) was as effective as selegiline (10 mg/kg) which is a potent irreversible monoamine oxidase B inhibitor for PD therapy.

PGC-1α, a transcriptional coactivator that regulates mitochondrial biogenesis, plays a critical role in promoting the expression of ROS scavenging enzymes and reducing oxidative stress. It is reported recently that dopaminergic neurons were more vulnerable to MPTP in PGC-1α knockout mice [32, 33], and a negative correlation was found between the activation of PGC-1α and α-syn accumulation [34]. In addition, PGC-1α and its downstream genes expression are reduced in the brains of PD patients [35], suggesting a high risk of oxidative stress. Nrf2, a nuclear transcription factor, is an attractive target for attenuating oxidative stress. According to previous studies, Nrf2 overexpression ameliorates neurodegeneration in PD drosophila model [36], while Nrf2 deficiency aggravates α-syn associated protein aggregation [37]. Currently, the molecular interaction between PGC-1a and Nrf2 has not yet been completely elucidated. However, PGC-1a and Nrf2 coordinate a large part of the enzymatic antioxidant defense system. Growing evidence indicates that PGC-1α/Nrf2 pathway is a promising target for drug discovery against PD [35, 38, 39]. Recently, Zhang et al. reported that fucoidan can protect the dopamine system in PD rats, likely mediated by reserving mitochondrial dysfunction via the PGC-1α/Nrf2 pathway [39]. In the present study, we confirmed that SDA could significantly increase the PGC-1α and Nrf2 expression in PC12/α-syn cells model as well as in the MPTP-induced PD animal model, indicating that neuroprotective effect of SDA may be partly mediated by reducing oxidative stress via upregulating antioxidant proteins and enhancing mitochondrial respiratory function through the PGC-1α/Nrf2 pathway.

Considering the diversity of the pathogenesis of PD, multi-targeting drugs acting on multiple molecular targets can be employed to address several pathological factors [40, 41]. Traditional Chinese herbal remedies a hope in addressing these complex pathological aspects by combining different drug molecules with different structures and mechanisms, acting on multiple malfunctioning targets and biological processes that cause the chronic and progressive neurodegeneration observed in PD [42, 43]. Activating PGC-1α/Nrf2 as the therapeutic target is also attractive since it affects multiple processes that are implicated in the pathogenesis of PD, especially in the regulation of oxidative stress. These findings presented in the current study will promote the research and development of SDA as a potential therapeutic agent in PD.

## Conclusions

In conclusion, our findings demonstrated that SDA had neuroprotection effect in dopaminergic PC12 cells induced with 6-OHDA. SDA had also displayed efficient protection to dopaminergic neuronal and alleviation to motor behavior properties in MPTP-induced PD mice. In PC12/α-syn cells and MPTP-induced PD animal models, SDA was highly efficacious in α-syn clearance associated with the activation of antioxidant PGC-1α/Nrf2 signal pathway, indicating it a potential therapeutic drug for PD.

## Data Availability

Please contact corresponding authors for data requests.

## Abbreviations

SDA: Shende’an tablet
α-syn: α-Synuclein
6-OHDA: 6-Hydroxydopamine
MPTP: 1-Methyl-4-phenyl-1,2,3,6-tetrahydropyridine
PGC-1α: Peroxisomeproliferator-activated receptor-γ coactivator-1α
Nrf2: Nuclear factor erythroid-2-related factor 2
SNpc: Substantia nigra pars compacta
UPS: Ubiquitin–proteasome system
ALP: Autophagy-lysosomal pathway
Dox: Doxycycline
Sele: Selegiline
Rap: Rapamycin
CQ: Chloroquine
DOPAC: 3,4-Dihydroxyphenylacetic acid
HVA: Homovanillic acid
