# Correction: Neuroprotective effects of Shende’an tablet in the Parkinson’s disease model

**DOI:** 10.1186/s13020-024-00965-3

**Published:** 2024-08-13

**Authors:** Xiaoyan Sheng, Shuiyuan Yang, Xiaomin Wen, Xin Zhang, Yongfeng Ye, Peng Zhao, Limin Zang, Kang Peng, Enming Du, Sai Li

**Affiliations:** 1 Nursing Department, Integrated Hospital of Traditional Chinese Medicine, Southern Medical University, Guangzhou, 510315 Guangdong China; 2 Department of Pharmacy, Guangdong Second Provincial General Hospital, Guangzhou, 510317 Guangdong China; 3 The Centre of Preventive, Integrated Hospital of Traditional Chinese Medicine, Southern Medical University, Guangzhou, 510315 Guangdong China; 4 Department of Pharmacy, Integrated Hospital of Traditional Chinese Medicine, Southern Medical University, Guangzhou, 510315 Guangdong China; 5 Department of Pharmacy, The Fifth Affiliated Hospital, Sun Yat-Sen University, Zhuhai, 519000 Guangdong China; 6 Zhengzhou Yihe Hospital of Henan University, Zhengzhou, 450047 Henan China; 7 Henan Eye Institute, Henan Eye Hospital, Henan Key Laboratory of Ophthalmology and Visual Science, People’s Hospital of Zhengzhou University, Henan University, School of Medicine, Henan Provincial People’s Hospital, Zhengzhou, 450003 Henan China

**Correction to: Chin Med (2021) 16:18** 10.1186/s13020-021-00429-y

Following publication of the original article [1], the authors reported that there were inaccuracies in the subimages of Figs. 1c, 2a, 3a, and the HO-1 and β-actin protein bands in Fig. 6. They have corrected these errors with the accurate subimages in the correct versions. These corrections do not alter the outcomes or conclusions of their study.

The correct Figs. 1, 2, 3 and 6 have been provided in this Correction.

The incorrect Fig. 1 is:

**Fig. 1:**
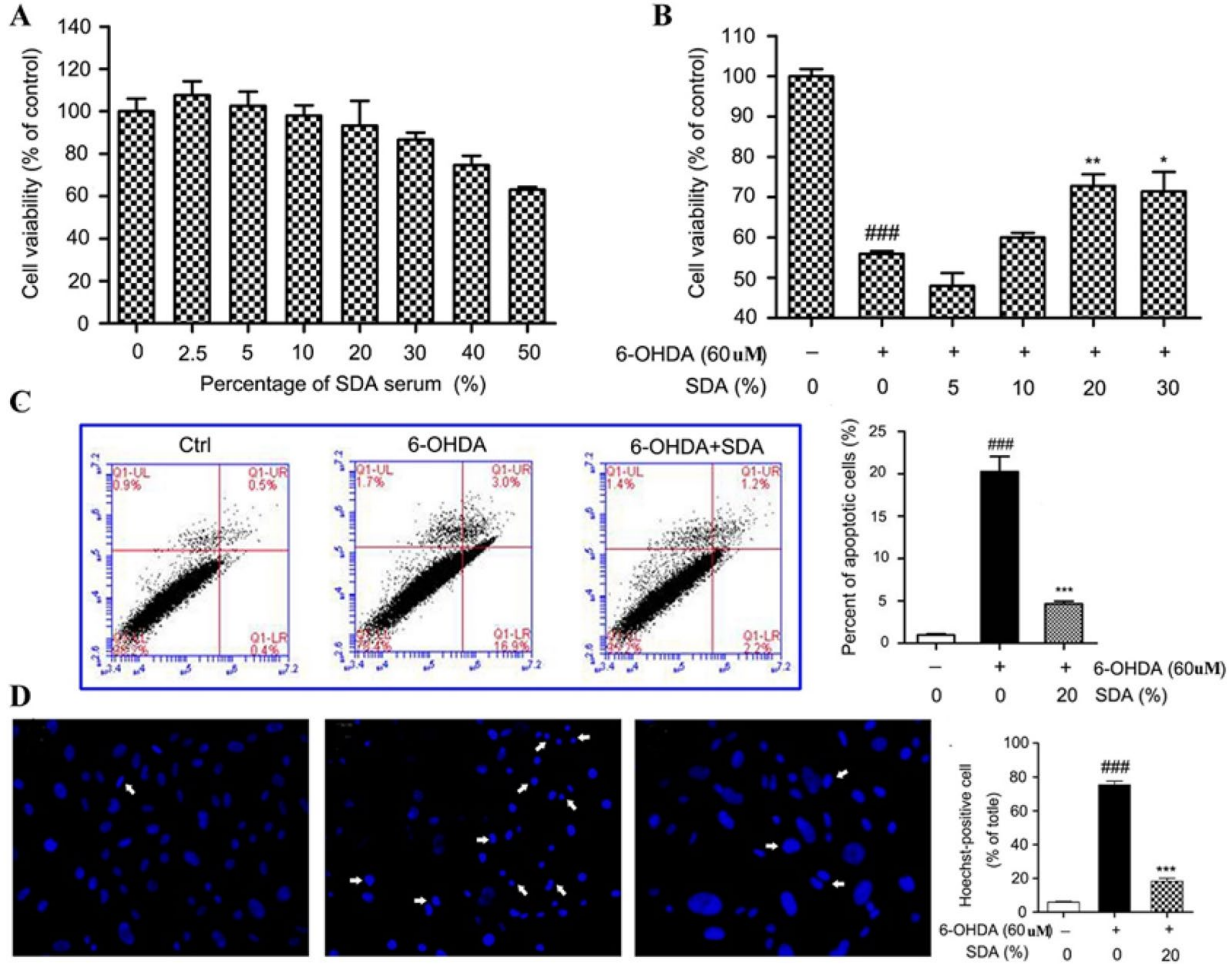
Neuroprotection of SDA rat serum against 6-OHDA-induced toxicity in PC12 cells. **a** The dose-dependent effect of SDA rat serum on the viability of PC12 cells; Neuroprotection of SDA rat serum against 6-OHDA-induced toxicity in PC12 cells was measured by CCK-8 assay (**b**), flow cytometry assay (**c**), and Hoechst staining. **d** The values are presented as the mean ± SEM from three independent experiments (^*###*^* P* < 0.001 compared to control group; *P < 0.05, **P < 0.01 and ***P < 0.001 compared to 6-OHDA group)

The correct Fig. 1 is:

**Fig. 1:**
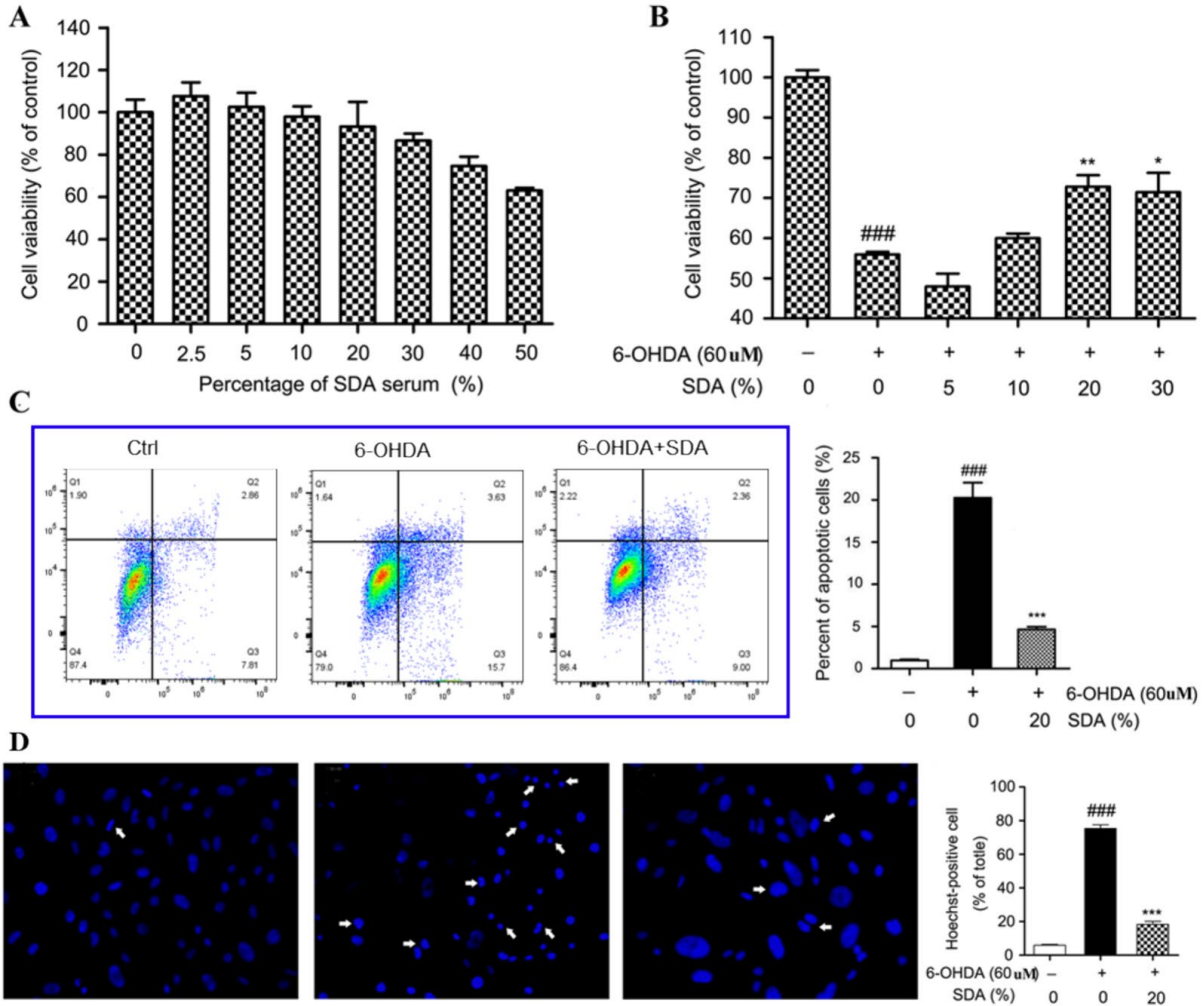
Neuroprotection of SDA rat serum against 6-OHDA-induced toxicity in PC12 cells. **a** The dose-dependent effect of SDA rat serum on the viability of PC12 cells; Neuroprotection of SDA rat serum against 6-OHDA-induced toxicity in PC12 cells was measured by CCK-8 assay (**b**), flow cytometry assay (**c**), and Hoechst staining. **d** The values are presented as the mean ± SEM from three independent experiments (^*###*^*P* < 0.001 compared to control group; *P < 0.05, **P < 0.01 and ***P < 0.001 compared to 6-OHDA group)

The incorrect Fig. 2 is:

**Fig. 2:**
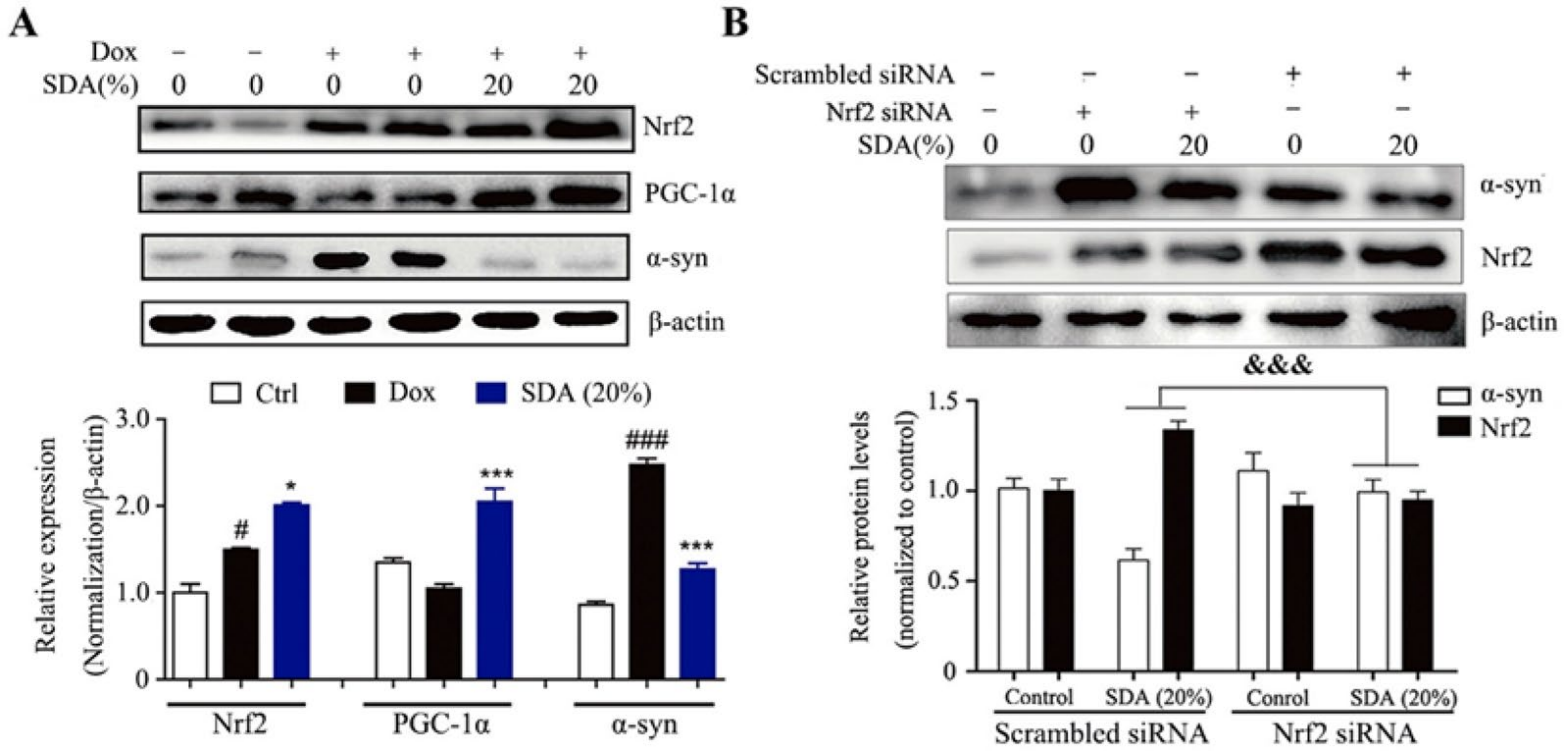
SDA promotes the α-syn clearance through activation of PGC-1α/Nrf2 signaling. **a** Representative immunoblots and densitometry data for α-syn, Nrf2 and PGC-1α in the inducible PC12/α-syn cells treated with doxycycline (Dox) followed by SDA; **b** Representative immunoblots and densitometry data for Nrf2 and α-syn levels in the inducible PC12/α-syn cells transfected Nrf2 siRNA or scrambled siRNA. Data from three independent experiments were expressed as mean ± SEM (^*#*^ *P* < 0.05, ^*###*^ *P* < 0.001 compared to control group; *P < 0.05, ***P < 0.001 compared to Dox-treated group; ^*&&&*^*P* < 0.001)

The correct Fig. 2 is:

**Fig. 2:**
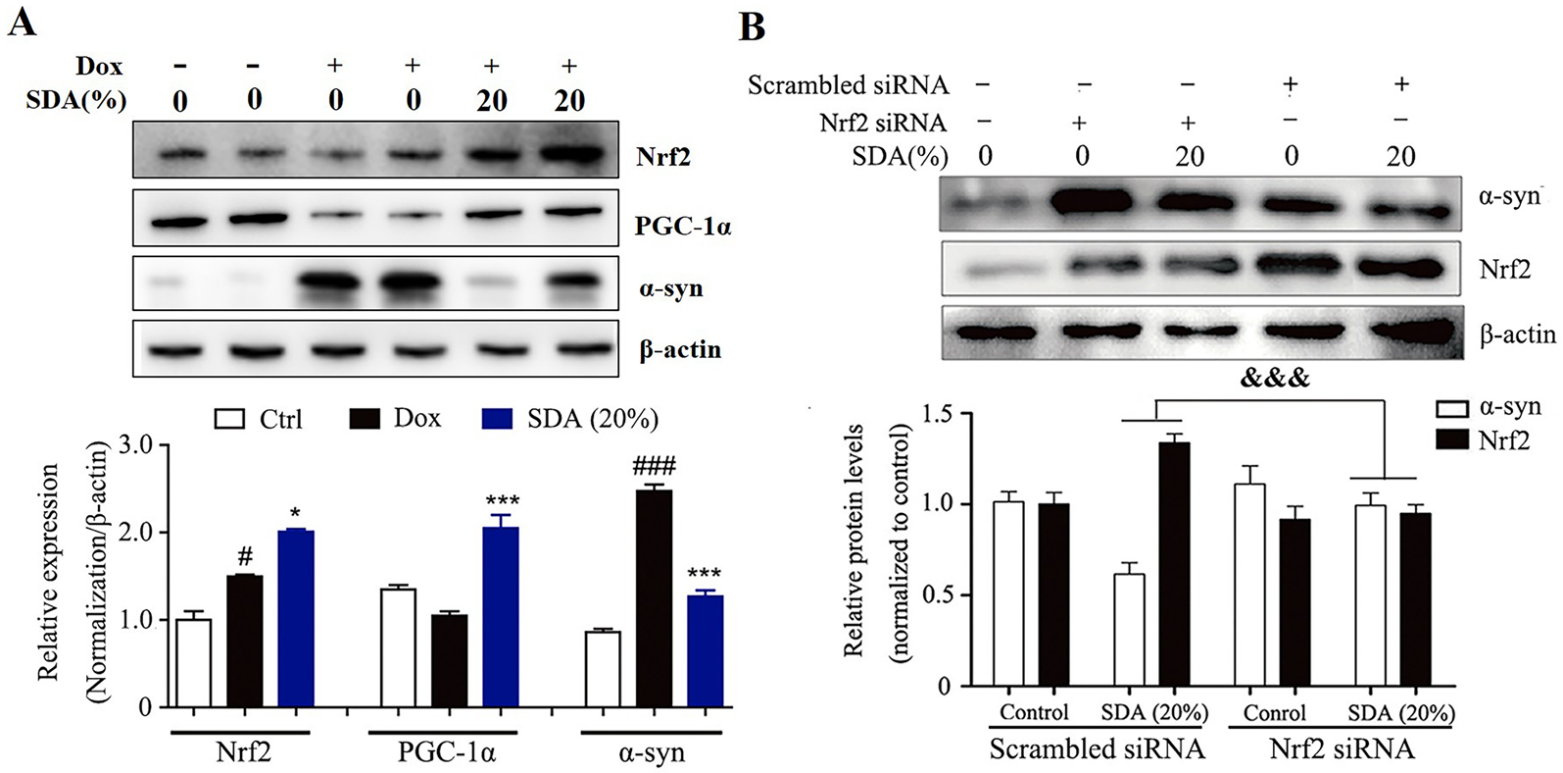
SDA promotes the α-syn clearance through activation of PGC-1α/Nrf2 signaling. **a** Representative immunoblots and densitometry data for α-syn, Nrf2 and PGC-1α in the inducible PC12/α-syn cells treated with doxycycline (Dox) followed by SDA; **b** Representative immunoblots and densitometry data for Nrf2 and α-syn levels in the inducible PC12/α-syn cells transfected Nrf2 siRNA or scrambled siRNA. Data from three independent experiments were expressed as mean ± SEM (^*#*^*P* < 0.05, ^*###*^*P* < 0.001 compared to control group; *P < 0.05, ***P < 0.001 compared to Dox-treated group; ^*&&&*^*P* < 0.001)

The incorrect Fig. 3 is:

**Fig. 3:**
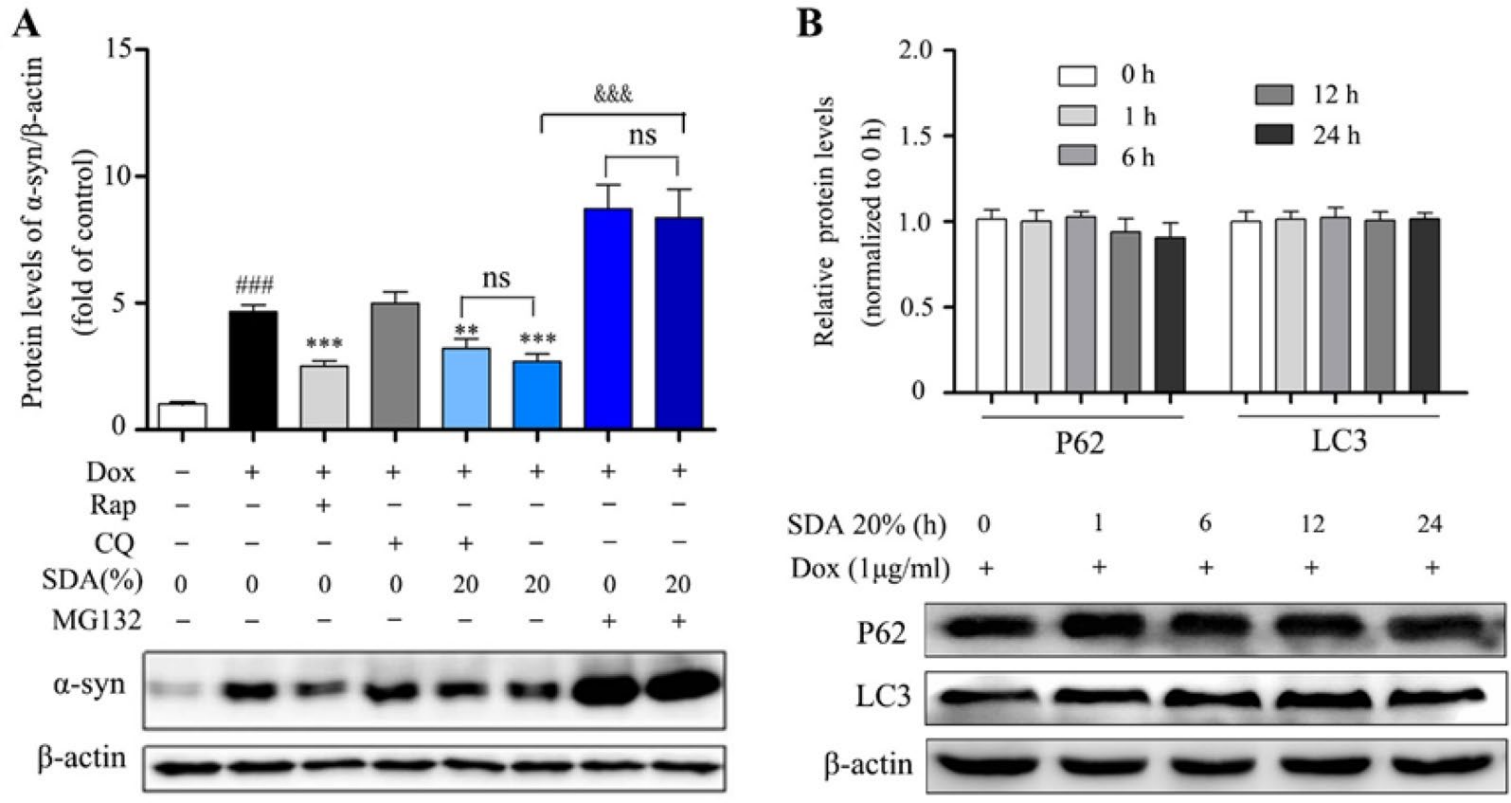
SDA promotes the α-syn clearance regulated by UPS pathway and independent of ALP pathway. **a** Representative immunoblot and quantification of α-syn levels in the inducible PC12/α-syn cells treated with Dox followed by 20% SDA, 20 µM autophagy inhibitor CQ, 0.7 µΜ proteasome inhibitor MG132 or 0.2 µM mTOR inhibitor Rap for another 24 h; **b **Representative immunoblots and quantification of p62 and LC3 levels in the inducible PC12/α-syn cells treated with Dox followed by SDA. Data from three independent experiments were expressed as mean ± SEM (^*###*^*P* < 0.001 compared to control group; **P < 0.01, ***P < 0.001 compared to Dox-treated group; ^*&*^P < 0.05, ^*&&&*^*P* < 0.001)

The correct Fig. 3 is:

**Fig. 3:**
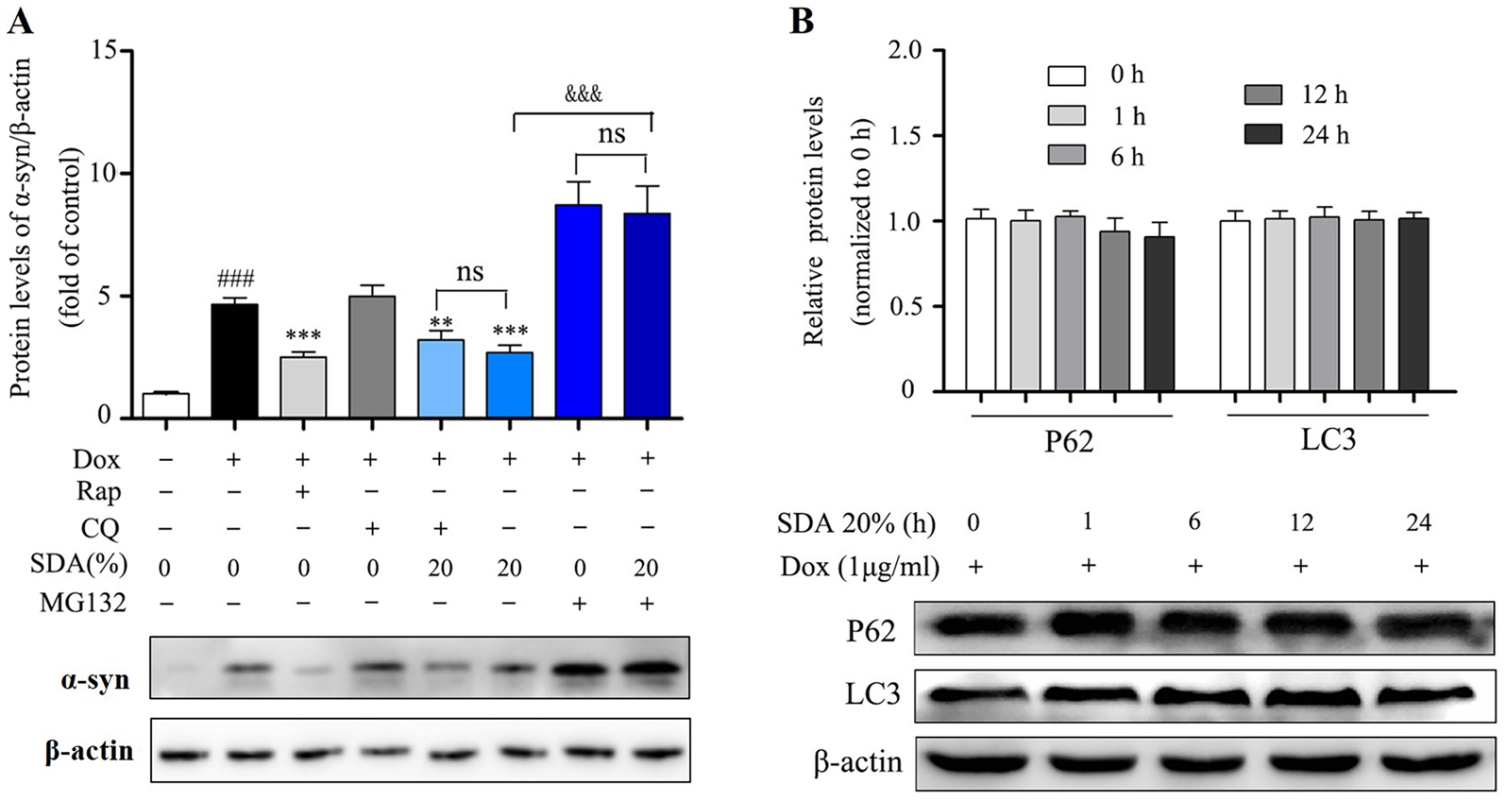
SDA promotes the α-syn clearance regulated by UPS pathway and independent of ALP pathway. **a** Representative immunoblot and quantification of α-syn levels in the inducible PC12/α-syn cells treated with Dox followed by 20% SDA, 20 µM autophagy inhibitor CQ, 0.7 µΜ proteasome inhibitor MG132 or 0.2 µM mTOR inhibitor Rap for another 24 h; **b** Representative immunoblots and quantification of p62 and LC3 levels in the inducible PC12/α-syn cells treated with Dox followed by SDA. Data from three independent experiments were expressed as mean ± SEM (^*###*^*P* < 0.001 compared to control group; **P < 0.01, ***P < 0.001 compared to Dox-treated group; ^*&*^P < 0.05, ^*&&&*^*P* < 0.001)

The incorrect Fig. 6 is:

**Fig. 6:**
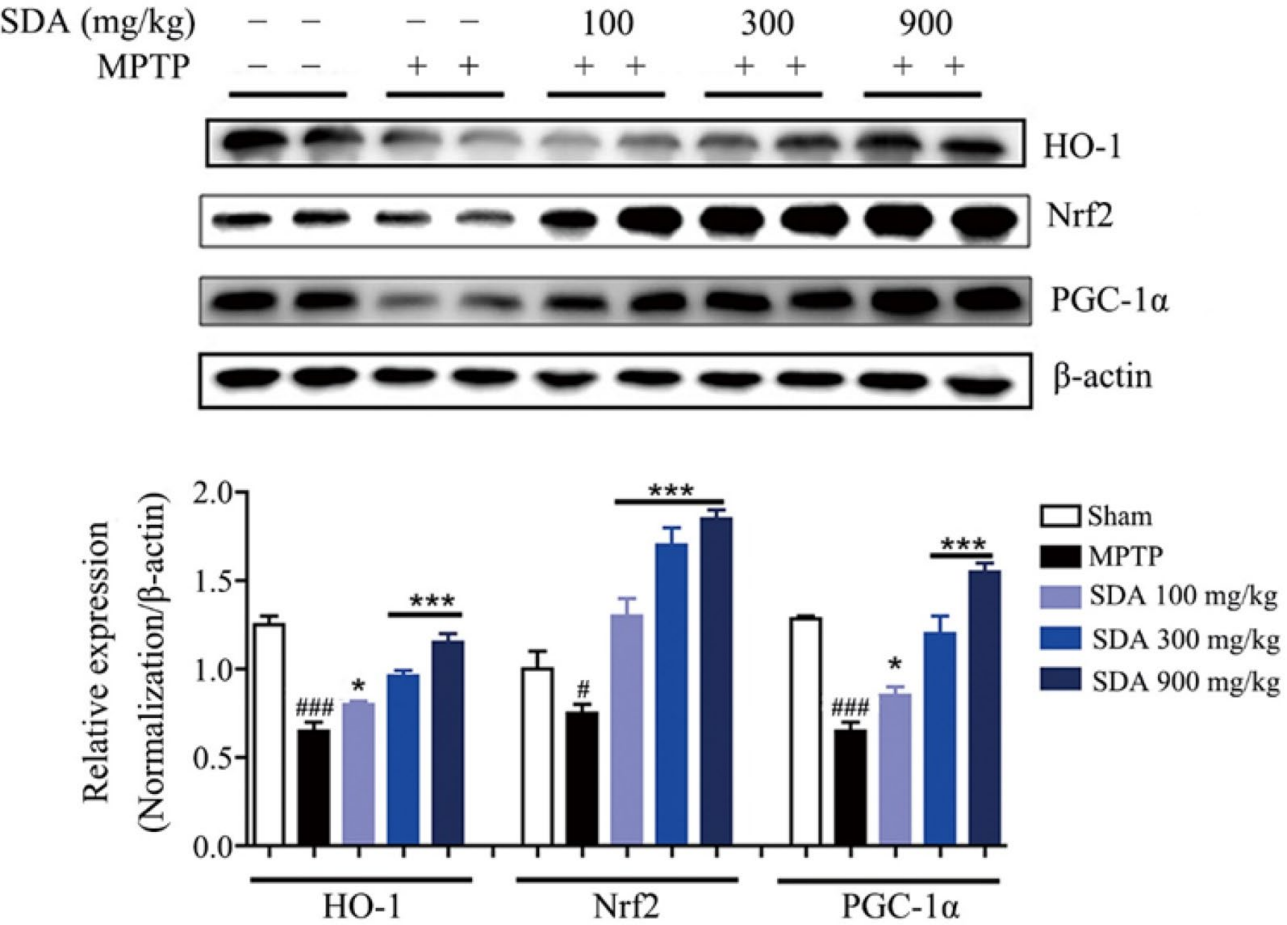
SDA activates PGC-1α/Nrf2 pathway to prevent neurodegeneration in MPTP-induced mice. Representative immunoblots and quantification of HO-1, Nrf2 and PGC-1α in the SNpc of MPTP-induced mice. Data were expressed as mean ± SEM. ^*#*^*P* < 0.05 and ^*###*^*P* < 0.001 compared to sham group; *P < 0.05 and ***P < 0.001 compared to MPTP group. n = 3/group

The correct Fig. 6 is:

**Fig. 6:**
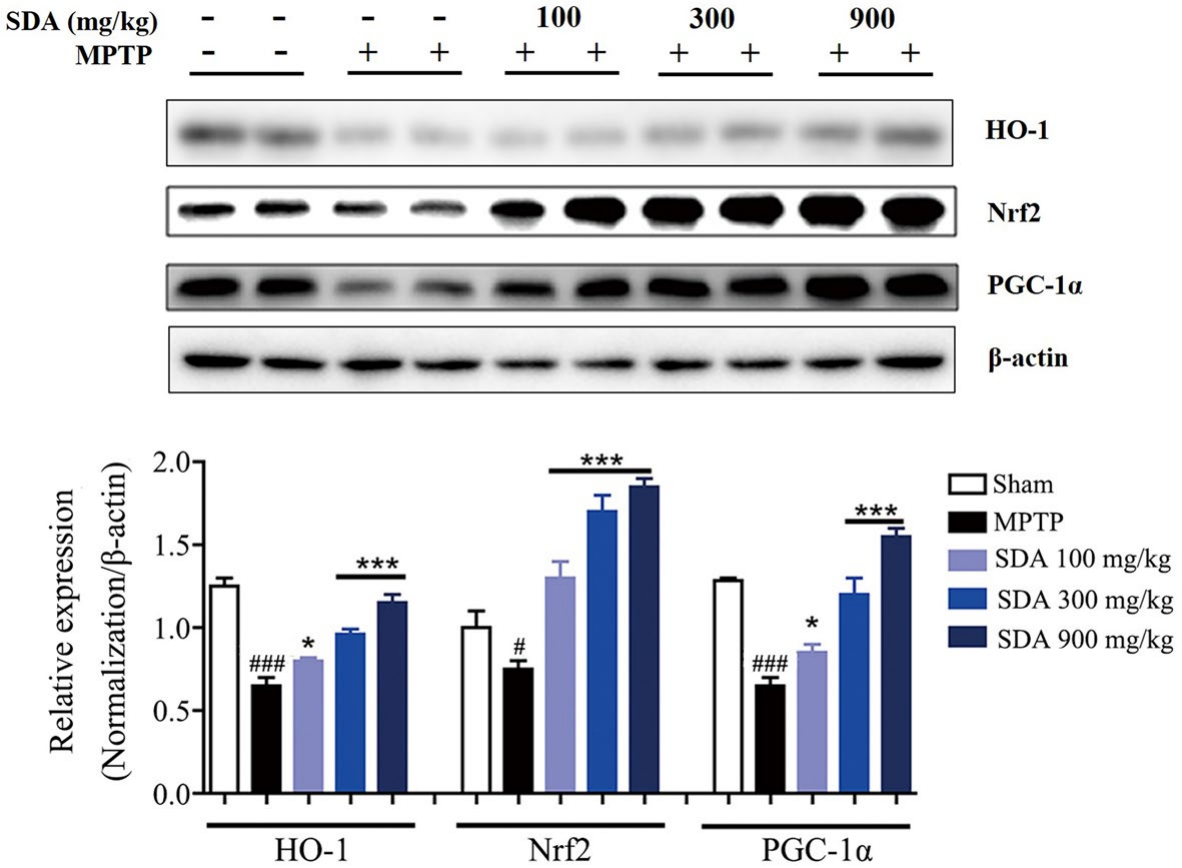
SDA activates PGC-1α/Nrf2 pathway to prevent neurodegeneration in MPTP-induced mice. Representative immunoblots and quantification of HO-1, Nrf2 and PGC-1α in the SNpc of MPTP-induced mice. Data were expressed as mean ± SEM. ^*#*^*P* < 0.05 and ^*###*^*P* < 0.001 compared to sham group; *P < 0.05 and ***P < 0.001 compared to MPTP group. n = 3/group

The original article [1] has been corrected.
